# Plasma lipids, tumor parameters and survival in HCC patients with HBV and HCV

**DOI:** 10.15761/jts.1000421

**Published:** 2020-09-16

**Authors:** H Akkiz, BI Carr, V Guerra, R Donghia, K Yalçın, U Karaoğullarından, E Altıntaş, A Özakyol, H Şimşek, HY Balaban, A Balkan, A Uyanıkoğlu, N Ekin, A Delik

**Affiliations:** 1 Çukurova University, Adana, Turkey; 2 İnonu University, Malatya, Turkey; 3 National Institute of Gastroenterology, S. de Bellis Research hospital, Castellana Grotte (BA), Italy; 4 Dikle University, Diyarbakır, Turkey; 5 Mersin University, Mersin, Turkey; 6 Eskişehir Osmangazi University, Eskişehir, Turkey; 7 Hacettepe University, Ankara, Turkey; 8 Gaziantep University, Gazientep, Turkey; 9 Harran University, Şanlıurfa, Turkey

**Keywords:** HCC, lipids, tumor size, aggressiveness, survival

## Abstract

**Introduction and aims:**

Hepatocellular carcinoma (HCC) is a consequence of chronic liver disease, particularly from hepatitis B or C and increasingly from obesity and metabolic syndrome. Since lipids are an important component of cell membranes and are involved in cell signaling and tumor cell growth, we wished to evaluate the relationship between HCC patient plasma lipids and maximum tumor diameter and other indices of HCC human biology.

**Methods:**

We examined prospectively-collected data from a multi-institutional collaborative Turkish HCC working group, from predominantly HBV-based patients, for plasma lipid profiles, consisting of triglycerides, total cholesterol, LDL-cholesterol (LDL) and HDL-cholesterol (HDL) and compared these with the associated patient maximum tumor diameter (MTD), portal vein thrombosis, alpha-fetoprotein (AFP) and also with patient survival.

**Results:**

We found that both low HDL (p=0.0002) and high LDL (p=0.003) levels were significantly associated with increased MTD, as well as in a final multiple linear regression model on MTD. The combination of low HDL combined with high HDL levels were significant in a regression model on MTD, PVT and an HCC Aggressiveness Index (Odds Ratio 12.91 compared to an Odds Ratio of 1 for the reference). Furthermore, in a Cox regression model on death, the HDL plus LDL combination had a significantly higher Hazard Ratio than the reference category.

**Conclusions:**

Low plasma HDL, high plasma LDL and especially the combination, were significantly related to more aggressive HCC phenotype and the combination was significantly related to a higher Hazard Ratio for death.

## Introduction

Alterations in blood lipid profiles and metabolism have been described in association withclinical hepatocellular carcinoma (HCC) [1–19]. Furthermore, lipid abnormalities have been described during the processes of hepatocarcinogenesis and in experimental rodent HCC and hepatoma cell lines and conversely, lipid administration can alter HCC biology [20–27]. Increasing trends in global obesity rates have focused on obesity-associated HCC [28,29].

In the present study, we report an analysis of baseline blood lipid parameters from a large cohort of HBV- and HCV-based HCC patients with baseline tumor and survival information. We show an association between lipid profile parameters with HCC growth parameters and survival.

## Methods

**Data collection:** we analyzed prospectively-collected data from our Turkish multi-institutional collaborating HCC working group [30,31], containing information on survival, baseline clinical and tumor characteristics on 1584 HCC patients who had full baseline tumor parameter data, including CT scan information on maximum tumor diameter (MTD), number of tumor nodules and presence of PVT; full blood counts; routine blood liver function tests, (total bilirubin, GGTP, albumin) and serum alpha-fetoprotein (AFP) values and who had lipid profile data. Patient demographic and survival information was also collected and recorded. Database management conformed to legislation on privacy and this study conforms to the ethical guidelines of the Declaration of Helsinki. Approval for this retrospective study on de-identified HCC patients was obtained from the Institutional Review Board of the participating centers.

Aggressiveness index: Aggressiveness Index [32,33] for Table 3 was calculated as the sum of scores: MTD (in tertiles): MTD<4.5; 4.5 ≤ MTD ≤ 9.6; MTD>9.6; for scores 1, 2, 3 respectively; AFP (cut-off): AFP<100; 100 ≤ AFP ≤ 1000; AFP>1000; for scores 1, 2, 3 respectively;PVT (No/Yes): PVT(No); PVT(Yes); for scores 1, 3 respectively; Number of Tumor Nodules: Nodules ≤ 3; Nodules>3; for scores 1, 3 respectively.

### Statistical methods

Characteristics of HCC patients were described by using mean (±SD) or proportions (%) for continuous or categorical variables respectively. For analytic purposes some variables were dichotomized: Glycemia (<100 or ≥100 mg/dL); Total Cholesterol (<200 or ≥200 (mg/dL), LDL Cholesterol (<160 or ≥160 mg/dL), HDL Cholesterol (>50(F),>40(M) or <50(F), <40(M) mg/dL) and Tryglycerides (<150 or ≥150 mg/dL). Differences between means were tested by using Wilcoxon rank-sum whereas proportions test was used to test differences for categorical variable. Multiple linear regression on dichotomized variables was applied to the data. To explore differences in metabolic parameters an Aggressiveness Index was built. The index reflects the sum of each single score as follows: MTD (in tertiles): MTD<4.5; 4.5≤MTD≤9.6; MTD>9.6; scores 1, 2, 3 respectively; AFP (cut-off): AFP<100; 100≤AFP≤1000; AFP>1000 ng/ml; scores 1, 2, 3 respectively; PVT (No/Yes): PVT (No); PVT (Yes); scores 1, 3 respectively; Tumor Nodules (number): Nodules≤3; Nodules>3; scores 1, 3 respectively. For survival analysis, the HDL Cholesterol ≥50(F), ≥40(M) or <50(F), <40(M) mg/mL and LDL Cholesterol <160 or >160 mg/mL were used. Cox’s Model was then fitted to the data. The proportional hazard assumption was evaluated by means of Schoenfeld residuals (SRT). Model fitting was evaluated by means of Akaike Information Criteria (AIC) and Bayesian Information Criterion (BIC). Risk estimators are expressed as Hazard Ratios (HR) and 95% Confidence Interval (95%CI).

Final Multiple Linear regression model of Aggressiveness Index score was used in the backward stepwise method, and all variables were examined as categorical. All statistical analyses were performed by using Stata statistical software, version STATA 16, 2019 (StataCorp LP, College Station, Texas).

## Results

### Lipid component levels in relation to tumor characteristics

Each of the major blood lipid components of this study were dichotomized as previously (REF) and the dichotomized subgroups were examined with respect to the 4 major tumor characteristics MTD, percent of patients with PVT, tumor multifocality and blood AFP levels (Table 1).Patients in the 2 glycemia level subgroups showed no significant difference in tumor characteristics. Likewise, for the triglyceride subgroups. However, patients in the HDL subgroups had significant differences in MTD only, with low HDL being associated with larger tumors, but not in any other tumor characteristic. Total cholesterol subgroups differed significantly in their AFP levels only. However, LDL subgroups had significant differences in 2 out of 4 of their tumor characteristics, namely in MTD and AFP levels (p=0.003 and 0.05 respectively), with percent of patients with PVT approaching significant differences p=0.08). A linear regression model of MTD was then constructed, on the single lipid variables.

Significance was found for HDL and LDL (Table 2), as suggested from the results in Table 2, for low HDL levels compared with high levels, and for high LDL levels compared with low levels (Table 2A). In a final regression model on all parameters in the backward stepwise method (Table 2B), both low HDL (β of 1.36, p<0.001) and high LDL (β of 2.28, p=0.007) were significantly different from their reference categories.

### Linear regression models of tumor characteristics and Cox model on death

Table 1 showed that low HDL or high LDL levels were associated with larger. Tumors diameters. We therefore considered HDL and LDL combined, in a linear or logistic regression model ofthe tumor parameters MTD, PVT and Tumor Aggressiveness index on HDL and LDL combined (Table 3). We found that compared to the reference category (high HDL and low LDL) of 1, that low HDL and high LDL combined yielded the highest β for MTD (β of 3.20), and the highest Odds Ratio (OR) for PVT (OR = 3.04) and Aggressiveness Index (OR = 12.91), with respect to any other combination of HDL and LDL. The data was then applied to a Cox regression model on death, considering LDL or HDL alone, or combined (Table 4). Significant differences in the Hazard Ratios (HRs) were found for HDL alone, HR of 1.29; LDL alone, HR of 1.24; and for the combination of low HDL and high LDL, with an HR of 1.54, compared to the reference category.

## Discussion

Changes in plasma lipid profiles have been well documented in patients having many cancers types, including HCC [2,3,8,11–19,34]. There seems to b e a 2-way process, since the presence of HCC is associated with plasma lipid changes-above, but microenvironmental changes in lipids can alter HCC content and biology [21, 23–26]. A possible mechanism for the lipidomic changes in HCC cells and HCC patient plasma profiles might be the well-described Warburg phenomenon in cancer cells [35,36], who first showed the preferential use by tumor cells of anaerobic glycolysis in glucose metabolism, leading to higher oxidative phosphorylation in cancer cells, which can result in increased products of lipid metabolism, such as very low density lipoproteins (VLDL) in HCC in associated with an increase in the lipolytic pathway, with an associated increase in ketone bodies, such as acetoacetate [37–39]. Furthermore, urinary metabolites of HCC patients showing increased free fatty acid metabolism, have also been suggested to be a sensitive and useful assay for HCC diagnosis and screening [40]. Also, strategies have recently been considered, for altering tumor lipid profiles as a therapeutic approach to modulating HCC biology [27,41–43].

The main findings of the current work are the association of low plasma HDL and high plasma LDLlevels with indices of tumor aggressiveness, both singly and especially together, both in relation to each lipid component that was considered, as well as in a linear regression model on MTD (Table 2). This was especially the case in a linear regression model on MTD or PVT or the Aggressiveness Index, when the 2 LDL components HDL and LDL were considered together. For the model on the Aggressiveness Index, the Odds Ratio increased from 1 for the reference HDL plus LDL combination to 12.91 for the combination of low HDL plus high LDL (Table 3). Increases in the Hazard Ratios were also found in the Cox model on death (Table 4), but the changes were less great than the changes in the odds ratios for tumor characteristics. There is little literature on the relationships of lipid components to tumor characteristics as shown in this work, although there are many papers on their relationships to survival and HCC recurrence post therapy [13–19,44]. Sometimes reports can be contradictory on the changes in lipoprotein levels and their prognostic significance in HCC. Thus, decreased HDL predicted a poorer prognosis in HCC [14], as did higher LDL levels [15] in 2 Chinese studies. In another Chinese liver transplant study however, lower HDL and LDL levels were reported [13]. However, in a Japanese report, low LDL was associated with increased mortality from HCC [16]. Multiple HCC studies show a decrease in both plasma HDL and LDL [45]. Plasma apolipoproteins are thought to be mainly decreased in HCC and bind to lipids to form lipoproteins that transport lipids through the blood stream. Of these, Apolipoprotein-AI (ApoAI) is the major protein component of plasma high-density lipoprotein (HDL) [46]. There is thus general agreement that lower lipoprotein levels occur in HCC patients, although their significance is not always clear. Interestingly, it there has recently been reported to be an association between GGT and HDL and both seem associated with fatty liver disease [47,48]. In the current report, although a majority of our HCC patients were HBV-based, we found little difference in the ratios of the lipid components between our HBV, HCV and non-hepatitis based HCC patients (data not shown).

## Conclusion

The current report shows an association of decreased plasma HDL and increased LDL levels, with both increased tumor parameters of aggressiveness- MTD and PVT, as well as with changes in survival. The central roles of lipids in tumor cell growth are leading to increased interest in manipulation of lipids as an approach to HCC therapy [25,27,42,43].

## Figures and Tables

**Table 1.** HCC patient Tumor Parameters according to plasma lipid dichotomizations.

	Glycemia (mg/dL)		
Variables^ψ^	<100	≥100	p^#^
MTD (cm)	6.27±4.20	5.78±3.80	0.12
PVT (%)	106 (30.55)	152 (28.90)	0.60^^^
AFP (IU/mL)	7357.48±53572.41	4409.28±28221.06	0.89
Multifocality (n ≥2) (%)	130 (35.52)	190 (36.12)	0.85^^^
	HDL (mg/dL)		
Variables^ψ^	[>=50 (F)]/[>=40 (M)]	[<50 (F)]/[<40 (M)]	p^#^
MTD (cm)	4.84±3.23	6.15±4.16	0.0002
PVT (%)	55 (24.77)	119 (30.43)	0.13^^^
AFP (IU/mL)	3350.67±28023.36	5334.98±22288.50	0.26
Multifocality (n ≥2) (%)	58 (25.66)	115 (29.34)	0.32^^^
	Triglycerides, (mg/dL)		
Variables^ψ^	<150	≥150	p^#^
			
MTD (cm)	5.73±3.89	6.11±3.94	0.30
PVT (%)	168 (28.57)	29 (27.36)	0.80^^^
AFP (IU/mL)	4115.46±30473.16	9271.83±36193.91	0.43
Multifocality (n ≥2) (%)	188 (31.92)	32 (28.57)	0.48^^^
	Total Cholesterol (mg/dL)		
Variables^ψ^	<200	≥200	p^#^
			
MTD (cm)	5.81±3.85	5.73±4.02	0.92
PVT (%)	166 (27.85)	32 (31.37)	0.48^^^
AFP (IU/mL)	4449.74±31465.38	7667.18±31443.45	0.03
Multifocality (n ≥2) (%)	189 (31.61)	35 (33.02)	0.78^^^
	LDL Cholesterol (mg/dL)		
Variables^ψ^	<160	≥160	p^#^
			
MTD (cm)	5.65±3.89	7.19±4.23	0.003
PVT (%)	162 (26.91)	17 (40.48)	0.08^^^
AFP (IU/mL)	4219.79±24481.65	6622.77± 14009.49	0.05
Multifocality (nr ≥2) (%)	177 (29.30)	18 (40.00)	0.16^^^

ψ All values: M±SD or No. of Patients (%),

# Wilcoxon rank-sum (Mann-Whitney) test;

^ Test for proportions; (HDL: High Density Lipoprotein Cholesterol; LDL, Low Density Lipoprotein Cholesterol; AFP, alpha-fetoprotein; MTD, Maximum Tumor Diameter; PVT, portal vein invasion (percent of patients)

**Table 2.** Linear regression model of MTD (cm), on single variables Glycemia, HDL, Triglycerides, Total Cholesterol, and LDL, all in categories (A). Final Multiple Linear regression model, in stepwise method, of MTD (cm), on Glycemia, HDL, Triglycerides, Total Cholesterol, and LDL, all in categories and included together in the model (B). Abbreviations: β: coefficient; se (β): standard error of coefficient; MTD, Maximum Tumor Diameter; HDL, High Density Lipoprotein Cholesterol; LDL, Low Density Lipoprotein Cholesterol; AFP, Alpha-Fetoprotein; F, Female; M, Male

	β	se(β)	p	95% C.I.
				
**(A)**				
Glycemia (mg/dL)				
<100 (*Ref. category*)	--			
≥100	−0.45	0.36	0.21	−1.15 to 0.26
HDL (ng/mL)				
[≥50(F)]/[≥40(M)] (*Ref. category*)	--			
[<50(F)]/[<40(M)]	1.49	0.36	<0.001	0.78 to 2.19
Triglycerides (mg/dl)				
<150 (*Ref. category*)	--			
≥150	0.24	0.47	0.61	−0.69 to 1.17
Total Cholesterol (mg/dL)				
<200 (*Ref. category*)	--			
≥200	−0.29	0.48	0.54	−1.25 to 0.66
LDL Cholesterol (mg/dL)				
<160 (*Ref. category*)	--			
≥160	1.55	0.69	0.03	0.19 to 2.90
				
**(B)**				
HDL (ng/mL)				
[≥50(F)]/[≥40(M)] (*Ref. category*)	--			
[<50(F)]/[<40(M)]	1.36	0.36	<0.001	0.65 to 2.07
Total Cholesterol (mg/dL)				
<200 (*Ref. category*)	--			
≥200	−1.01	0.58	0.08	−2.16 to 0.14
LDL Cholesterol (mg/dL)				
<160 (*Ref. category*)	--			
≥160	2.28	0.83	0.007	0.64 to 3.92
			

**Table 3.** Linear regression model of MTD, PVT and Aggressiveness Index score as categorical. All models on HDL and LDL categories combined.

				MTD (cm)				PVT (No/Yes)				Aggressiveness Index score (4/≥5)			
Parameter				β	se(β)	p-value	95% C.I.	OR	se(OR)	p-value	95% C.I.	OR	se(OR)	p-value	95% C.I.
															
HDL*	&	LDL^¥^													
High		Low	(*Ref. category*)	1				1				1			
High		High		1.25	1.24	0.31	−1.19 to 3.70	1.14	0.79	0.85	0.29 to 4.45	1.08	0.78	0.92	0.26 to 4.43
Low		Low		1.25	0.33	<0.001	0.60 to 1.91	1.25	0.25	0.26	0.85 to 1.84	1.11	0.21	0.56	0.77 to 1.61
Low		High		3.20	0.77	<0.001	1.68 to 4.72	3.04	1.29	0.009	1.32 to 6.97	12.91	13.31	0.01	1.71 to 97.42
															

* HDL (mg/dL): High (0), [≥50(F)]/[≥40(M)]; Low (1), [<50(F)]/[<40(M)];

¥ LDL (mg/dL): Low (0), <160; High (1), ≥160. Abbreviations: β, coefficient; se (β), standard error of coefficient; OR, Odds-Ratio; se (OR), standard error of Odds-Ratio; MTD, Maximum Tumor Diameter; PVT, Portal Vein Thrombosis; HDL, High Density Lipoprotein Cholesterol; LDL, Low Density Lipoprotein Cholesterol. Aggressiveness Index as sum of scores: MTD (in terciles): MTD<4.5; 4.5≤MTD≤9.6; MTD>9.6; scores 1, 2, 3 respectively; AFP (cut-off): AFP<100; 100≤AFP≤1000; AFP>1000 ng/ml; scores 1, 2, 3 respectively; PVT (No/Yes): PVT (No); PVT (Yes); scores 1, 3 respectively; Tumor Nodules (number): Nodules≤3; Nodules>3; scores 1, 3 respectively

**Table 4.** Cox regression model on single parameters in the model.

Parameter	HR	se(HR)	p	95% C.I.
HDL (mg/dL)*				
High (Ref. category)	1			
Low	1.29	0.13	0.02	1.05 to 1.58
LDL (mg/dL)^¥^				
High (Ref. category)	1			
Low	1.24	0.21	0.20	0.89 to 1.72
HDL, LDL Combined				
HDL (High) & LDL (Low) (Ref. category)	1			
HDL (High) & LDL (High)	1.12	0.39	0.74	0.57 to 2.21
HDL (Low) & LDL (Low)	1.27	0.14	0.03	1.02 to 1.58
HDL (Low) & LDL (High)	1.54	0.33	0.04	1.01 to 2.36

* HDL (mg/dL): High (0), [≥50(F)]/[≥40(M)]; Low (1), [<50(F)]/[<40(M)];

¥ LDL (mg/dL): Low (0), <160; High (1), ≥160; Abbreviations: HR, Hazard-Ratio; se (HR), standard error of Hazard-Ratio; HDL Cholesterol, High Density Lipoprotein; LDL Cholesterol, Low Density Lipoproteins

## Abbreviations:

HCC: Hepatocellular Carcinoma
NAFLD: Non-Alcoholic Fatty liver disease
MTD: Maximum Tumor Diameter
AFP: Alpha-Fetoprotein
PVT: Portal Vein Thrombosis
HDL: High Density Lipoprotein
LDL: Low Density Lipoprotein
HBV: Hepatitis B virus
HCV: Hepatitis C Virus
CT: Computerized Axial Tomography
MRI: Magnetic Resonance Imaging
